# Workers dominate male production in the neotropical bumblebee *Bombus wilmattae *(Hymenoptera: Apidae)

**DOI:** 10.1186/1742-9994-8-13

**Published:** 2011-06-08

**Authors:** Anett Huth-Schwarz, Adolfo León, Rémy Vandame, Robin FA Moritz, F Bernhard Kraus

**Affiliations:** 1 Institut für Biologie, Martin-Luther-Universität Halle-Wittenberg, Germany; 2 Department of Community Ecology, Helmholtz Centre for Environmental Research (UFZ), Halle, Germany; 3 Universidad de Ciencias y Artes de Chiapas, Tuxtla Gutiérrez, Chiapas, Mexico; 4 El Colegio de la Frontera Sur, San Cristóbal de las Casas, Chiapas, Mexico; 5 Department of Zoology and Entomology, University of Pretoria, South Africa

## Abstract

**Background:**

Cooperation and conflict in social insects are closely linked to the genetic structure of the colony. Kin selection theory predicts conflict over the production of males between the workers and the queen and between the workers themselves, depending on intra-colonial relatedness but also on other factors like colony efficiency, sex ratios, cost of worker reproduction and worker dominance behaviour. In most bumblebee (*Bombus*) species the queen wins this conflict and often dominates male production. However, most studies in bumblebees have been conducted with only a few selected, mostly single mated species from temperate climate regions. Here we study the genetic colony composition of the facultative polyandrous neotropical bumblebee *Bombus wilmattae*, to assess the outcome of the queen-worker conflict over male production and to detect potential worker policing.

**Results:**

A total of 120 males from five colonies were genotyped with up to nine microsatellite markers to infer their parentage. Four of the five colonies were queen right at point of time of male sampling, while one had an uncertain queen status. The workers clearly dominated production of males with an average of 84.9% +/- 14.3% of males being worker sons. In the two doubly mated colonies 62.5% and 96.7% of the male offspring originated from workers and both patrilines participated in male production. Inferring the mother genotypes from the male offspring, between four to eight workers participated in the production of males.

**Conclusions:**

In this study we show that the workers clearly win the queen-worker conflict over male production in *B. wilmattae*, which sets them apart from the temperate bumblebee species studied so far. Workers clearly dominated male production in the singly as well the doubly mated colonies, with up to eight workers producing male offspring in a single colony. Moreover no monopolization of reproduction by single workers occurred.

## Background

In the majority of eusocial hymenopteran species the queen is typically the only fertile female that produces both diploid female and haploid male offspring. Despite this reproductive dominance of the queen, workers in many species still retain a considerable reproductive capacity. Although they are normally sterile and unable to mate, they occasionally activate the ovaries to produce haploid male offspring [1]. Worker reproduction is an important reproductive pathway whenever the colony loses the queen and cannot replace the gyne. In fact, worker reproduction can be found in all larger taxa of the eusocial hymenoptera, in ants, wasps and bees, including the bumblebees (Bombini) [2-6]. Bumblebees are typically monogynous and monandrous [7] and kin selection theory predicts a conflict between queen and workers over male production [8-10]. Workers should prefer the production of their own sons instead of raising the male offspring of their mother queen. In bumblebees, this queen-worker conflict is best studied in *Bombus terrestris*, where the queen dominates male production and successfully suppresses worker reproduction [11-14]. With the onset of the last stage in the colony life cycle, the so called competition phase, the males are produced and the queen and workers exhibit aggressive behavior towards each other (e.g. destroying egg shells, egg eating, buzzing, attacking, matricide) to maintain or gain reproductive dominance [14,15]. In spite of this change in worker behaviour the worker male parentage remains modest with less than five percent [11,13]. One particular reason for low worker reproduction is the so called policing behavior, where workers destroy the eggs laid by other workers, often in association with aggressive behavior of laying as well as non-laying workers towards each other [14,15].

In general, kin selection theory predicts worker policing to be primarily adaptive in multiple mated species, if intracolonial relatedness is below the critical threshold of *r *= 0.5 and the effective mating frequency is larger than two. However, worker policing and the absence of worker produced males in general, also occur in single mated species, like in the hornet *Vespa crabro *[16] or in the ant *Camponotus floridanus *[17] and thus also other factors besides relatedness seem to be of importance. Among these factors are colony level costs of worker reproduction and interaction with the queen-worker conflict over the sex ratio [5]. A model developed by Ohtsuki and Tsuji [18] indicates that worker policing and reproduction depends also on colony growth and development stage. Further also queen policing of worker laid eggs can suppress the reproduction of workers in single mated species with small colony sizes, like it is the case in the paper wasps *Polistes dominulus *[19] and *Polistes chinensis antennalis *[20] and in the Bumblebee *B. terrestris *[12].

Most bumblebee species studied so far are singly mated with only few exceptions [21-23,7], and show a low to moderate degree of worker reproduction [e.g. [11,24-27]]. Moreover the vast majority of studies have been conducted with temperate species. Here we study the neotropical bumblebee *B. wilmattae*, which has an annual life cycle and colony sizes of up to several hundred individuals. Moreover *B. willmattae *is one of the few bumblebee species that are known to be facultative polyandrous [23] and we used this species to detect potential worker policing in colonies with singly or multiply mated queens, to study the outcome of the queen-worker conflict over male production using microsatellite genotyping.

## Results

All analyzed microsatellite loci were highly polymorphic with an average of 6.8 ± 2.7 alleles per locus resulting in observed heterozygosity of 0.96 ± 0.09 (Table 1). A total of 116 workers (*n_w_*,Table 2) were used to determine the queen mating frequency (*m_obs_*) within each colony (Table 2). The overall effective mating frequency was 1.21 ± 0.31 as two colonies (C1 and C4) were double mated and skewed paternities occurred (C4: χ^2 ^= 4.96; p = 0.03). The intracolonial relatedness (*g_ww_*) among the workers inferred from the parental generation was on average 0.68 ± 0.09.

**Table 1:** Population genetic parameters of the analyzed colonies.

	B100	B124	B126	B131	B132	population
*An*	10	4	8	8	4	6.8 ± 2.68
*range*	162 - 184	254 - 274	152 - 166	118 - 146	158 - 162	118 - 274
*H_o_*	1.00	0.80	1.00	1.00	1.00	0.96 ± 0.09

The number of alleles (*An*), range and size of the microsatellite fragments in bp and observed heterozygosity (*H*_o_) per locus and population based on the data obtained from females over all five *B. wilmattae *colonies.

**Table 2:** Genetic colony structure of the five *B. wilmattae *colonies.

Colonies	collecting date	*N_w _*/*N_m_*	m_obs_*	*n_w_*	*n_m_*	*n_pw _*+*nde*	*p_Ae_*
C1	14 Dec 2005	75/67	2	24	24	4 + 0.09	0.125
C2	04 Dec 2008	314/243	1	23	24	7 + 0.74	0.031
C3	10 Dec 2005	345/470	1	24	22	6 + 0.18	0.250
C4	23 Nov 2005	52/120	2	22	21	6 + 0.49	0.250
C5	19 Nov 2005	141/145	1	23	24	7 + 0.26	0.063

The collecting date of the analyzed colonies, number of workers (*N_w_*) and males (*N_m_*) recorded either after the death of the queen (C1 and C3-5) or directly after excavating and dissecting (C2), number of observed matings (*m_obs_*) [*[19]], number of genotyped workers (*n_W_*) and males (*n_m_*), estimated number of workers producing own male offspring (*n_pw_*) corrected for the non-detection error (*nde*) based on a Poisson distribution and probability of assigning worker offspring as queen offspring (*p_Ae_*)

**Figure 1:**
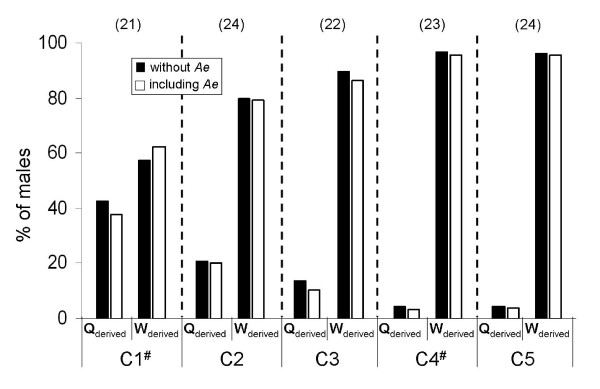
**Male parentage in the five analyzed colonies of the neotropical bumblebee *Bombus wilmattae***. Shown is the proportion of males, which were either queen (Q) or worker (W) derived in the single as well as double mated (#) colonies. The proportion of male offspring is given without correction for assignment error (*Ae*; black bars) and with correction for assignment error (white bars). The numbers of analyzed males are shown in brackets.

A total of 115 male genotypes could be used in the analyses (*n_m_*,Table 2) and an average of 82.8% +/- 15.9% per colony could be unambiguously assigned as worker offspring. Correcting these values for the assignment error, an average of 84.9% +/- 14.3% males per colony were worker produced (Figure 1). In the colonies with doubly mated queens 62.5% (C1) and 96.7% (C4) of the males were produced by workers. Based on the genotypes of worker-produced males, the number of reproducing workers ranged from four to eight across colonies (Table 2).

The proportion of males produced by workers of the two patrilines in the colonies with the doubly mated queens was not significantly skewed (χ^2 ^= 3.46; p = 0.62), although the more frequent patrilines represented about 2/3 of the genotyped males in our sample (C1 = 66.7%; C4 = 65.2%).

## Discussion

Our results clearly indicate that the vast majority of the male offspring in the neotropical bumblebee *B. wilmattae *is worker produced. Given that at least four out of five colonies were definitely queen right at the point of male sampling, matricide, which occurs in several annual species of social insects [28,14], can be excluded as cause for the high percentage of worker produced males. Thus apparently in *B. wilmattae*, the workers win the queen-worker conflict over male production, with about 85% of all genotyped males estimated to be worker derived.

Offspring of up to eight workers could be detected among the genotyped males of a given colony and both patrilines participated in male production in the colonies with doubly mated queens. Since the average relatedness was not significantly below the threshold of r < 0.5 in any of the analyzed colonies worker policing would not have been expected to be adaptive based on kin selection theory alone. The presence of up to eight reproductive workers in each colony indicates that there is no monopolization of reproduction by single workers, but in the absence of behavioral data there is no definitive evidence against the presence of worker policing. The few dominant male producing workers might well police male eggs of other subordinate workers or of the queen. Based on the overall number of workers present during dissection of the colonies C1 and C3-C5, an estimated 3.91% of the workers of these colonies successfully produced male offspring that reached adult stage. In three queen right *B*. *terrestris *colonies, that were studied by van Doorn and Heringa [14], 13.1% of the workers (224.3) became egg-layers during the competition phase, but almost all eggs were eaten by the queen. Thus, just a very small percentage of males reach adult stage and a small amount of workers could successfully reproduce. Worker policing also occurs in monandrous and monogynous social hymenoptera like *B. terrestris *[14,15] or *Polistes chinensis antennalis *[20]. Besides relatedness at least five other factors [5], among them the cost of worker reproduction [4] and worker dominance behaviour [29], can favor the evolution of worker policing also in species with single mated queens and resulting high worker relatedness.

Our results are in accordance with another neotropical species, *B. atratus*, where Zucchi [25] found 90% of the males to be worker produced based on the observation of egg laying behaviour. Such worker dominance over male production seems absent in the various temperate *Bombus *species, that have been studied so far. Although Van Honk et al. [15] claimed that the queen loses her reproductive dominance after the competition point and workers start male production in *B. terrestris*, a subsequent study [14] based on a much larger sample of colonies showed that most males were actually queen produced. These results gained further support by studies applying microsatellite markers and genotyping [13,11], showing that 95.7% of the males were queen produced. Although 38% of the workers laid eggs, only very few of these eggs developed into adult males, showing that the queen wins the conflict over male production in spite of laying workers in queen right *B. terrestris *colonies. Also in other temperate bumblebee species the workers seem to lose the conflict over male parentage. For example, in four queen right colonies of *B. impatiens *only 9% of workers had developed ovaries and only two workers were observed to lay eggs which were later on destroyed by the queen [30]. Similar in *B. melanopygus *just 19% of males were worker derived [26], in *B. hypnorum *20% [24] and in *B. ignitus *5% [27]. In contrast worker reproduction was higher in *B. deuteronymus *with between 30% to 50% worker produced males [31]. Although workers do produce males after the death of the queen, this seems to be insufficient to offset the large number of queen produced males over the full season [15,26].

In our study sampling of males took place in the second half of the colony cycle two to four weeks before the death of the queen, where sampling ended and the colonies were dissected. As a consequence the eggs from which our sampled males had emerged, were laid five weeks to two months prior to the death of the queen and thus in her full presence. The only exception might be colony C2, where we cannot exclude the absence of the queen at the point of sampling; however also in colony C2 the males sampled had been produced three to four weeks earlier depending on the estimated development time of drones in bumblebees [11,32,30].

Clearly *B. wilmattae *deviates from the typical male production pattern found in many temperate bumblebee species. Four of the five analyzed colonies (C2-C5) showed remarkable high numbers of worker produced males (80% - 97%). Only in one colony (C1) the percentage of worker male offspring was less with 60%. Also, this was the colony with the lowest number of successfully reproducing workers, but still the worker produced males outnumbered those produced by the queen.

## Conclusions

In conclusion, we could show that tropical bumblebee *B. willmattae*, in spite of its colony life cycle similar to that of temperate species, strongly deviates from other *Bombus *species with regard to male production. The workers clearly dominated male production in the singly as well the doubly mated colonies and no monopolization of reproduction by a single worker occurred. Whether worker dominated male production is a common feature of tropical *Bombus *species, like the study by Zucchi [25] might indicate, or rather a rare variant within the genus *Bombus *is open to future studies employing molecular markers.

## Methods

### Species in focus

The bumblebee species *B. (Pyrobombus) wilmattae *Cockerell, 1912 is native to the tropical mountainous regions of southern Mexico (Chiapas) and Guatemala [33]. *B. wilmattae *has an annual life cycle, with queens independently founding the colonies, which can reach a size of several hundred individuals at peak season. The production of males occurs at the end of the colony cycle typically from end of October to January during dry season.

### Sampling

Four queen right colonies of *B. wilmattae *(C1 and C3-C5) were sampled from mid November until mid December 2005 and one colony (C2) in mid December 2008 (Table 2). All five colonies were collected in the vicinity of the village Unión Juárez, Chiapas, Mexico, close to the Guatemalan border (15° 3'52.31"N, 92° 4'52.32"W). The colonies sampled during 2005 were transported into the laboratory and further colony development was monitored there. The colonies were monitored in the lab until the death of the queen, which occurred in the last week of December (25.12. ± 2 days) after which the colonies were dissected. Worker and male samples were collected during the time window between transportation to the lab and the death of the queen, when dead individuals were found in the colonies during daily inspections. Samples from colony C2 were taken immediately after unearthing of the colony in the field. In case of colony C2 no queen could be retrieved either due to missing to catch her during the sampling process, or because the colony already lacked a queen. All samples were stored in 95% ethanol at -20°C until DNA extraction; the sampling dates are given in Table 2.

### Molecular analysis

A total of 24 workers and 24 males of each of the five colonies were used for genotyping. DNA was extracted from one leg of each individual following the *Chelex*-extraction protocol by Walsh et al. [34]. The sampled individuals were genotyped with five microsatellite markers [Table 1, [35,36]]. In colony C2 additionally four new developed markers of Stolle et al. [37] were used (data not shown) to obtain a sufficient resolution to discriminate between worker and queen produced males. All individuals were genotyped following standard polymerase chain reaction (PCR) protocols in an automated DNA capillary sequencer (MegaBACE 1000) according to manufacturer's instructions. Allele scoring was done using the MegaBase Fragment Profiler software.

### Data analysis

The origin of the sampled males (queen or worker produced) was determined using the inferred queen and father genotypes from the worker samples. Males with at least one allele in common with the fathering male of the colony could be unambiguously assigned as worker male offspring. Males that only had alleles of the queen were assigned as queen offspring. However, such males could still be worker derived, whenever all mother queens' alleles but none of the father's alleles had been transferred into a male produced by a worker. We calculated the probability of wrong assignment for each colony as follows *p_Ae _* = (1/2)^n(A)^, where n(A) is the number of informative alleles, and corrected the number of worker and queen produced males accordingly. The number of workers that produced the male offspring was estimated using the genotypes of the worker produced males and the program COLONY 1.2 [38]. The error for a non-detection of reproducing workers was assigned based on a Poisson distribution.

## Competing interests

The authors declare that they have no competing interests.

## Authors' contributions

AHS carried out the molecular analyses, data analyses and drafted the manuscript. FBK and RFAM participated in the interpretation of the molecular data, gave advice for statistical analyses and contributed to the writing of the manuscript. AL and RV provided the infrastructure for maintenance of the *Bombus wilmattae *colonies and performed sampling. All authors contributed to the writing of the manuscript and approved the final manuscript.
